# 

**Published:** 1908-07-18

**Authors:** 


### CONTENTS.

| | PAGE |
|---|---|
| **The Dignity of Medicine** ... | 405 |
| **The Admission of Women to the Royal College of Surgeons** ... | 406 |
| **Annotations**— | |
| The B.M.A. Meeting—Headache Powders—The Sleeping Sickness Controversy ... | 407 |
| **Medical Opinion and Movement** ... | 408 |
| **Hospital Clinics**— | |
| Paralysis in Children. By James Taylor, M.D., F.R.C.P. ... | 411 |
| Infective Endocarditis. By Theodore Fisher, M.D., F.R.C.P. | 413 |
| **Medicine**— | |
| The Varieties of Anæmia in Chronic Tuberculosis ... | 415 |
| A Chemical Theory of Scurvy ... | 416 |
| **Surgery**— | |
| Acute Retention of Urine—III. ... | 417 |
| Some Aspects of Intestinal Obstruction ... | 4.8 |
| **Ophthalmology**— | |
| Cyclitis and Irido-Cyclitis—I. ... | 419 |
| **Resident Medical Officers' Department**— | |
| Responsibility and Authority ... | 420 |
| **Dental Department**— | |
| The Prevalence and Cause of Dental Caries ... | 421 |
| **Toxicology**— | |
| Nutmeg Poisoning ... | 422 |
| **"The Hospital" Medical Book Supplement.—No. IX.** ... | 423 |
| **Hospital Administration**— | |
| Graduate Study on the Continent—Germany ... | 427 |
| Social and Poor-Law Problems ... | 428 |
| **News and Coming Events** ... | 429 |
| **Nursing Administration**— | |
| Nursing as a Collegiate Course ... | 431 |
| **The Graduates' Medical Schools** ... | 432 |
| **Editor's Letter-Box** ... | 432 |

